# Comparative genomics provides insight into maize adaptation in temperate regions

**DOI:** 10.1186/s13059-016-1020-2

**Published:** 2016-07-13

**Authors:** Matthew B. Hufford

**Affiliations:** Department of Ecology, Evolution, and Organismal Biology, Iowa State University, Ames, IA 50011 USA

## Abstract

A new study provides insights into the evolution of maize during its global spread into temperate regions from its origin in coastal Mexico.

Please see related Research article: http://genomebiology.biomedcentral.com/articles/10.1186/s13059-016-1009-x

## Adaptation during maize diffusion

Maize was domesticated along the tropical Pacific coast of southwest Mexico approximately 10,000 years before present [1]. Since its domestication, maize has spread from its center of origin into dramatically different habitats, a migration that required adaptation to novel environmental conditions. While the domestication center of maize is situated in a warm, low-elevation, short-day region with ample precipitation, maize has subsequently colonized the cool slopes of the Andes Mountains, the arid deserts of the American Southwest, and the temperate, long-day growing conditions of southern Canada. Presumably, farmer selection has favored maize that is well adapted to local conditions, creating a number of differentially evolved populations.

These farmer-driven evolutionary experiments have left signatures of selection and targeted loci that can be detected through genome-wide comparisons between materials adapted to various environmental conditions. Such studies provide basic insights into the process of crop evolution and have potential applications for modern breeding efforts that seek to adapt crops to environmental extremes. An investigation by Unterseer and co-authors [2] recently published in *Genome Biology* uses a comparative genomic approach to characterize unique adaptations in temperate Flint and Dent varieties of maize.

## History of Flint and Dent maize

The Northern Flint and Southern Dent landraces are the major germplasm pools for the maize varieties currently grown in temperate regions of the northern hemisphere. Northern Flint landrace kernels are characterized by a hard outer protective layer making them “as hard as flint”, whereas Southern Dents have a higher soft starch content that leads to a small indentation (or “dent”) at the crown of their kernels. At the time of European colonization, Northern Flints were found as far north as what is now southern Canada and were likely derived by Native Americans from maize in the American Southwest. Southern Dent landraces are thought to have been introduced early on into what is now the southeastern USA by the Spanish from Mexico [3]. While both the Flint and the Dent varieties were adapted to temperate conditions, including long days and short growing seasons, Flint was also exceptionally cold tolerant.

During the 19th century, farmers in the American Corn Belt found that maize varieties made of a mixture of Northern Flint and Southern Dent materials were heterotic, displaying superior fitness compared with their parental landraces. They were therefore widely adopted and became what are known as the Midwestern or Corn Belt Dents; the modern hybrid maize breeding program in the USA has drawn upon this admixed material. Genetic studies have shown, however, that the modern US heterotic groups have only a minor contribution from the Northern Flints and instead are largely dominated by the Southern Dent germplasm [3, 4]. In contrast, modern maize germplasm from central and northern Europe is heavily influenced by the Northern Flints [5]. The different histories of American and European temperate maize present an opportunity for a replicated study on adaptation to high-latitude conditions.

## Finding the genetic basis of temperate adaptation

In order to identify the genetic underpinnings of temperate adaptation and to investigate differentiation in Dent- and Flint-derived maize, Unterseer and co-authors genotyped a large panel of 136 founder lines for American and European breeding programs and found over 500,000 single nucleotide polymorphisms [2]. Candidate loci for adaptation in Flint and Dent were identified using multiple population genetic statistics capable of detecting selection and differentiation and by applying conservative thresholds for outlier loci. Hundreds of loci were detected that are potentially involved in Flint and Dent adaptation, including genes thought to underlie differences between these germplasm pools in herbivore defense, as well as the extreme cold tolerance of the Flints.

The authors focused primarily on a subset of candidate genes with known roles in flowering time (FT) since this trait is an important distinguishing factor between the two germplasm pools, with Flints flowering substantially earlier than Dents. The authors’ findings suggest that different components of the FT network were targeted during Flint and Dent evolution. Specifically, Dent FT candidates are weighted toward response to environmental factors such as light and photoperiod, whereas Flint candidates are involved primarily in endogenous pathways. By also assessing patterns of diversity and differentiation at FT loci in early Flint landraces and comparing them with modern elite Flint-derived lines, the authors demonstrated that much of the selection on these candidates had occurred prior to modern breeding efforts. Finally, in order to explore the phenotypic effects of their candidate FT loci, the authors utilized a maize library consisting of small Flint introgressions into a Dent background. Introgression lines with Flint haplotypes at Flint FT candidate genes showed significantly earlier male flowering than lines without introgression at these sites.

Candidates identified through scans for selection that have also been phenotypically validated are rare in the literature; the study therefore represents one of the few examples in which a bottom-up approach based on population genetics has made the link to phenotypic effects.

## The importance of comparative studies in crop evolution

A fruitful approach to evolutionary genomics during the era of next-generation sequencing has been the “evolve and resequence” strategy, in which a base population of an organism is subjected to a specific selection pressure for several generations and then both the base and the evolved populations are subsequently resequenced and compared [6]. Dramatic changes in allele frequencies over the course of the experiment at particular loci suggest these loci have played a role in adaptation to the particular selection pressure that has been applied. Such studies have revealed, for example, genomic regions underlying body size in *Drosophila melanogaster* [7] and toleration of high temperature in *Escherichia coli* [8].

The histories of many of the world’s most important crops have involved farmer-assisted diffusion away from a center of origin into vastly different habitats. These diffusion events can be seen as unintentional “evolve and resequence” experiments that can now be utilized to gain a basic understanding of the process of crop adaptation. Comparative genomic studies of these unintentional experiments should yield considerable insight. The work of Unterseer and colleagues published in *Genome Biology* demonstrates the utility of this comparative approach [2]. Other similar comparative genomics studies include recent assessments of high-elevation adaptation in maize [9] and Fusarium Head Blight resistance in an experimental population of barley [10]. Moving forward, such studies should continue to identify genetic loci underlying important crop adaptations. These loci, and the adaptations they confer, can then be more efficiently targeted during the process of crop improvement.

## Abbreviation

FT, flowering time.
